# Relationship Between Dairy Products Intake and Risk of Endometriosis: A Systematic Review and Dose-Response Meta-Analysis

**DOI:** 10.3389/fnut.2021.701860

**Published:** 2021-07-22

**Authors:** Xiangying Qi, Wenyan Zhang, Mingxiu Ge, Qiang Sun, Lei Peng, Wenke Cheng, Xuepeng Li

**Affiliations:** ^1^Department of Obstetrics and Gynecology, Affiliated Hospital of Jining Medical University, Zaozhuang Municipal Hospital, Zaozhuang, China; ^2^Department of Obstetrics and Gynecology, The Second Affiliated Hospital of Shandong University, Jinan, China; ^3^Department of Cardiology, Heart Center Leipzig at University Leipzig, Leipzig, Germany; ^4^Department of Gynecology, Affiliated Hospital of Jining Medical University, Zaozhuang Municipal Hospital, Zaozhuang, China

**Keywords:** dairy products, endometriosis, dose-response, milk, meta-analyses

## Abstract

**Objective:** Diet lifestyle can influence the risk of endometriosis. Therefore, we conducted a systematic meta-analysis to investigate the association between dairy products and the risk of endometriosis. Besides, we performed a dose-response meta-analysis to evaluate the amount of dairy intake affecting the risk of endometriosis.

**Methods:** Relevant studies were searched from Pubmed, Embase databases, Cochrane Library, and Web of Science from the inception to November 6th, 2020. Also, the dose-response meta-analysis was conducted. All the pooled results were performed by risk ratios (RRs).

**Results:** Finally, seven high-quality studies were included in the present meta-analysis. Total dairy intake was inversely associated with the risk of endometriosis, and the risk of endometriosis tended to decrease with a decrease in the risk of endometriosis when dairy products intake was over 21 servings/week (RR 0.87, 95% CI 0.76–1.00; *p*_non−linearity_ = 0.04). Similarly, people who consumed more than 18 servings of high-fat dairy products per week had a reduced risk of endometriosis (RR 0.86, 95% CI 0.76–0.96). When stratified-analyses were conducted based on specific dairy product categories, it indicated that people with high cheese intake might have a reduced risk of endometriosis (RR 0.86, 95% CI 0.74–1.00). Other specific dairy products such as whole milk (RR 0.90, 95% CI 0.72–1.12), reduced-fat/skim milk (RR 0.83, 95% CI 0.50–1.73), ice cream (RR 0.83, 95% CI 0.50–1.73), and yogurt (RR 0.83, 95% CI 0.62–1.11) have not shown significant evidence of an association with the risk of endometriosis. However, there is a higher risk of endometriosis in the females with high butter intake compared to females with low butter intake (1.27, 95% CI 1.03–1.55).

**Conclusions:** Overall, dairy products intake was associated with a reduction in endometriosis, with significant effects when the average daily intake ≥3 servings. When analyzed according to the specific type of dairy product, it was shown that females with higher high-fat dairy and cheese intake might have a reduced risk of endometriosis. However, high butter intake might be associated to the increased risk of endometriosis. More future studies are needed to validate and add to this finding.

## Introduction

Endometriosis was defined as a growing infiltrate of endometrial tissue (glands and mesenchyme) outside the endometrium that bleeds repeatedly to form nodules and masses causing pain and may invade any part of the body, such as the bladder, kidneys, ureter, lungs, pleura, affecting the quality of life (1). Endometriosis was a devastating disease for reproductive women. It was reported that endometriosis affected 5–15% of women of reproductive age (2), of whom 30–50% are infertile (3). Although the occurrence of endometriosis is considered to be associated with risk factors such as immune, endocrine, genetic, and anatomical disorders (4, 5), the etiology of the disease is not fully clear (6).

Diet is a highly controllable risk factor for many chronic diseases, but its contributing role to endometriosis has not been extensively explored (7). A literature review from Parazzini et al. (8) suggested that women with endometriosis appear to consume fewer vegetables and omega-3 polyunsaturated fatty acids while consuming higher amounts of red meat, coffee, and trans fats. Dairy products as an important part of the diet are rich in a variety of amino acids and high in calcium, making them an ideal nutritious food. Studies have shown that dairy products and dietary calcium intake are negatively correlated with oxidative and inflammatory stress (9–12). Besides, a high intake of dairy products might reduce vascular inflammation (9). Retrograde menstruation is postulated to be a potential causal catalyst for endometriosis, and the high magnesium levels found in dairy products would relax smooth muscle and might reduce retrograde menstruation (13). Therefore, some researchers have hypothesized that dairy products intake might reduce the risk of endometriosis, while based on the currently limited studies this hypothesis has not been well-established. To address these gaps, we conducted a systematic meta-analysis to investigate the association between dairy products and the risk of endometriosis. Besides, we performed a dose-response meta-analysis to evaluate the amount of dairy intake affecting the risk of endometriosis.

## Methods

### Search Strategy

The protocol and report of this meta-analysis were based on a meta-analysis of observational studies from the Guidelines on Epidemiology (MOOSE) (14). Relevant studies were searched from Pubmed, Embase databases, Cochrane Library, and Web of Science from the inception to February 7th, 2021. Also, a manual library search was conducted. Two groups of medical subject headings (MeSH), including “dairy products” and “endometriosis” were used to ensure a comprehensive search. In addition, previous meta-analyses and systematic reviews were reviewed for full inclusion in the study, if applicable. A detailed search strategy is provided in Appendix 1.

### Study Selection

The inclusion criteria for this study were as follows:

1. Dairy intake as the exposure of interests.
2. Control group was non-dairy intake or less frequent dairy intake.
3. The endpoint of the study was the occurrence of endometriosis.
4. Study type was restricted to case-control, cohort study or randomized controlled trial.
5. There were available data on the maximum adjustment risk ratios (RRs), odds ratios (ORs), hazard ratios (HRs), together with corresponding 95% confidence intervals (CIs) in the study. Exclusion criteria:

1. Studies on non-dairy intake.
2. The endpoint of the study was non-occurrence of endometriosis.
3. Cross-sectional studies were excluded.
4. Conference abstracts, letters, and case reports were excluded.

### Data Extraction and Quality Assessment

The following data were extracted using the Unified Data List, including first author, publication year, country, study design, sample, date of recruitment, age, dairy consumption, type of dairy, dairy consumption, and endometriosis ascertainment were also recorded. The maximum covariate-adjusted ORs, RRs, HRs were extracted. Any disagreements or disputes that arose during the data extraction process were resolved by mutual agreement. Besides, the study used the Newcastle-Ottawa Scale (NOS) (15) to assess the quality of the study, with an overall score of 9 points. Specifically, studies with a NOS score of more than 6 stars were considered high-quality studies, while studies with a NOS score of fewer than 6 stars were considered low-quality studies.

### Statistical Analysis

The first primary endpoint of this study was a qualitative analysis of the relationship between dairy product intake and the risk of endometriosis. In general, HR is equal to RR, which can be roughly considered as RR (16). ORs were also converted to RRs, RR = OR/[(1-P0) + (P0 × OR)], where P_0_ indicates the incidence of the outcome in the unexposed group (17). The corresponding 95% CI is: SElog(RR) = SElog(OR) × log(RR)/log(OR) (18). When the P_0_ was low (<10%), the odds ratio is very similar to the risk ratio (16). Therefore, all the data are expressed as RRs. Inter-study heterogeneity between studies was evaluated using the *I*^2^ statistic, where *I*^2^ values of 25, 50, and 75% indicate low, moderate, and high inconsistency, respectively. Besides, we performed subgroup analysis and meta-regression to explore potential sources of heterogeneity and to compare different groups. Sensitivity analyses were performed by eliminating one study at a time to examine its impact on the combined results. To estimate the combined RRs more conservatively, we used a random-effects model, as it better explained the heterogeneity between studies. In addition, publication bias was assessed by Begg's and Egger's test (19, 20).

The second endpoint of this study was to systematically assess the effect of dairy product intake on endometriosis. To this end, we conducted a quantitative dose-response meta-analysis. To maximize the available studies, we used the robust error meta-regression approach described by Xu and Doi (21) to establish a potential dose-response relationship between dairy product intake and the risk of endometriosis. In this “one-stage” framework approach, each included study was treated as a cluster across the whole population, which required that the studies include at least two categories. In this study, the restricted cubic spline was employed to fit the potential non-linear trend with three knots, and the non-linear *P*-value was calculated by testing the second spline coefficient of zero. The non-linear model was adopted when *P* for non-linear ≤ 0.05; otherwise, the linear model was adopted. In general, the included studies needed to take the category of lowest dose as the reference, and when the study of the non-lowest dose was taken as the reference, we converted it through the Excel macro file made by Hamling et al. (22) based on Greenland and Longnecker's theory (23). When the number of cases in a category was missing, the original authors were contacted. Besides, when studying open intervals, the amplitude was assumed to be the same as the adjacent category (24). Referring to the Food Frequency Questionnaire (FFQ) scale, it is generally considered that one serving is 250 ml. Also, all doses are expressed in the form of servings/week for the convenience of calculation. All data were analyzed using Stata 12.0.

## Results

**Figure 1:**
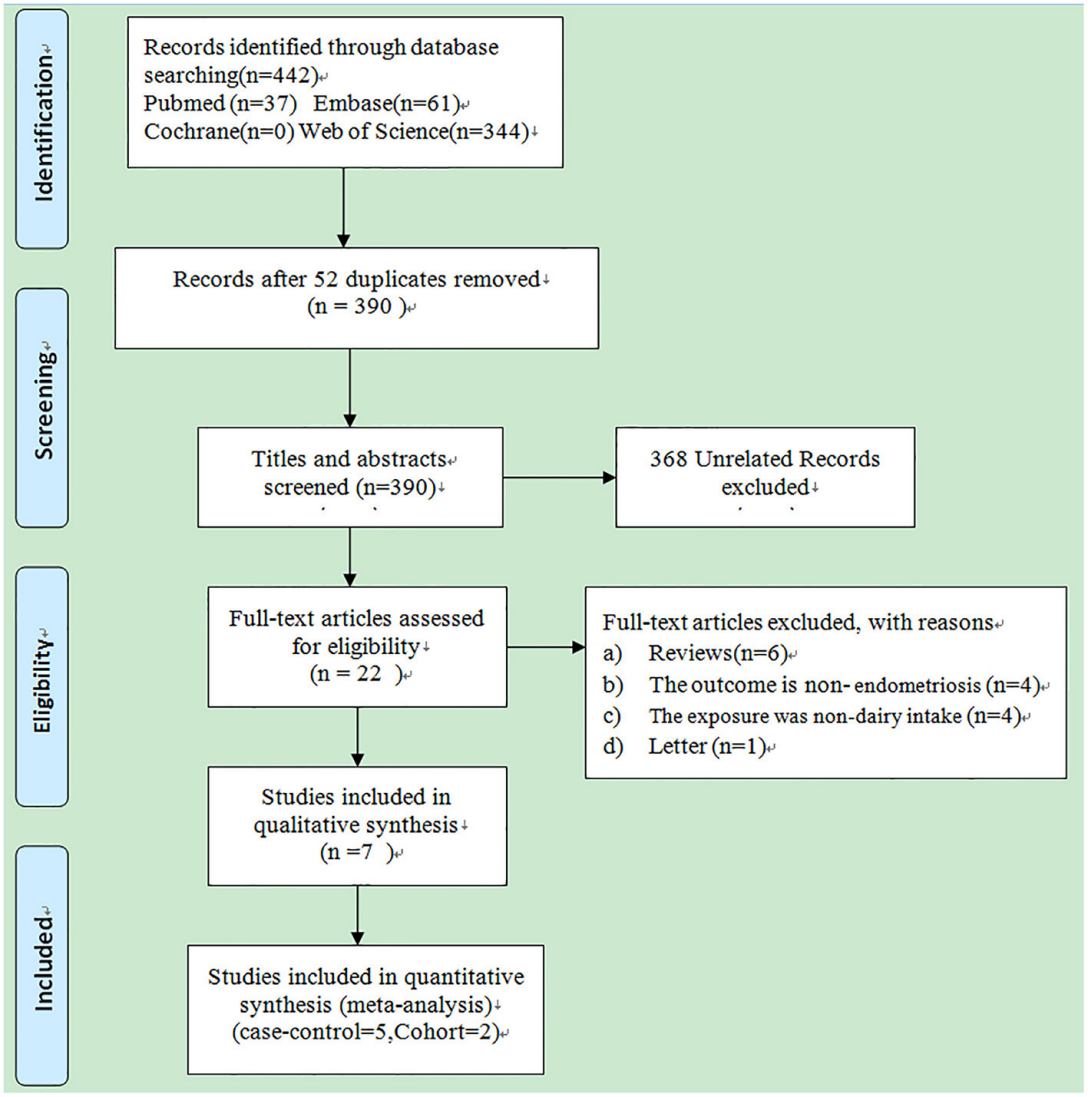
Flow chart of the study retrieval process.

A total of 442 studies were searched from four electronic databases, PubMed, Embase, Cochrane library, and Web of science, as shown in Figure 1. Other additional studies were not revealed by manual search. Of these 442 studies, 52 were excluded because of duplication, whereas 368 unrelated studies were deleted after screening the titles and abstracts. The remaining 22 studies were carefully read in full, of which 15 were excluded for the following reasons: (a) review (*n* = 6); (b). The outcome is non-endometriosis (*n* = 4); (c). The exposure was non-dairy intake (*n* = 4); (d) Letter (*n* = 1). Finally, seven observational studies (25–31) were included in the present meta-analysis, including 5 case-control studies and 2 cohort studies. Baseline characteristics of all included studies are shown in Table 1. Two of the seven included observational studies reported an association between whole milk, ice cream, yogurt, and reduced-fat/skim milk intake and the risk of endometriosis; three studies reported an association between low-fat dairy, high-fat dairy and butter intake and the development of endometriosis; four studies reported an association between total dairy intake and the risk of endometriosis; five studies reviewed an association between the intake of milk, cheese and the risk of endometriosis. For dose-response meta-analysis, four studies focused on the relationship between the consumption of milk, cheese and the occurrence of endometriosis; three studies focusing on the correlation between total dairy products intake and the development of endometriosis; two studies focusing on the association between high-fat dairy products consumption and the risk of endometriosis. The quality of the included studies is assessed in Supplementary Table 1. Of these 7 studies, 3 scored 8 stars; 4 scored 7 stars. All studies scored 6 stars or higher and were considered high-quality studies.

**Table 1:** The details of the included 7 observational studies.

**References**	**Country**	**Study design**	**Endometriosis cases**	**Age**	**Dairy consumption (servings)**	**Types of dairy**	**Dairy consumption**	**Endometriosis ascertainment**
Nodler et al. (22)	US	PC	581	Mean 41.3y	≤ 1/day, 2/day, 3/day, 4/day, >4/day	Total dairy foods, milk, butter, cheese, yogurt, ice-cream	HS-FFQ	Laparoscopy
Parazzini et al. (23)	Italy	CC	504	Median 33y	≤ 2, 3–5, ≥6/week	Milk, cheese, butter	A Structured Questionnaire	Laparoscopy
Samaneh et al. (24)	Iran	CC	78	31y	Q1, Q2, Q3, Q41	Total dairy foods	FFQ	Laparoscopy
Ashrafi et al. (25)	Iran	CC	207	31.5y	0, 1–6, ≥7/week	Milk, cheese	A Structured Questionnaire	Laparoscopy
Harris et al. (26)	US	PC	1,385	36.0y	≤ 2, 5–6/week, 1/day, 2/day, 3/day, >3/day	Total dairy foods, milk, butter, cheese, yogurt, ice-cream	FFQ	Laparoscopy
Trabert et al. (27)	US	CC	284	18–49y	≤ 1/day, 2/day, >2/day	Total dairy foods	FFQ	ICD-9
Heilier et al. (28)	Belgium	CC	88	Mean 33y	NA	Milk, butter, cheese	FFQ	Medical Records

*HS-FFQ, food frequency questionnaire about diet during high school; FFQ, Food Frequency Questionnaire; ICD-9, International Classification of Disease; NA, Not appliable*.

### Meta-Analysis

#### Total Dairy Product (High vs. Low)

**Figure 2:**
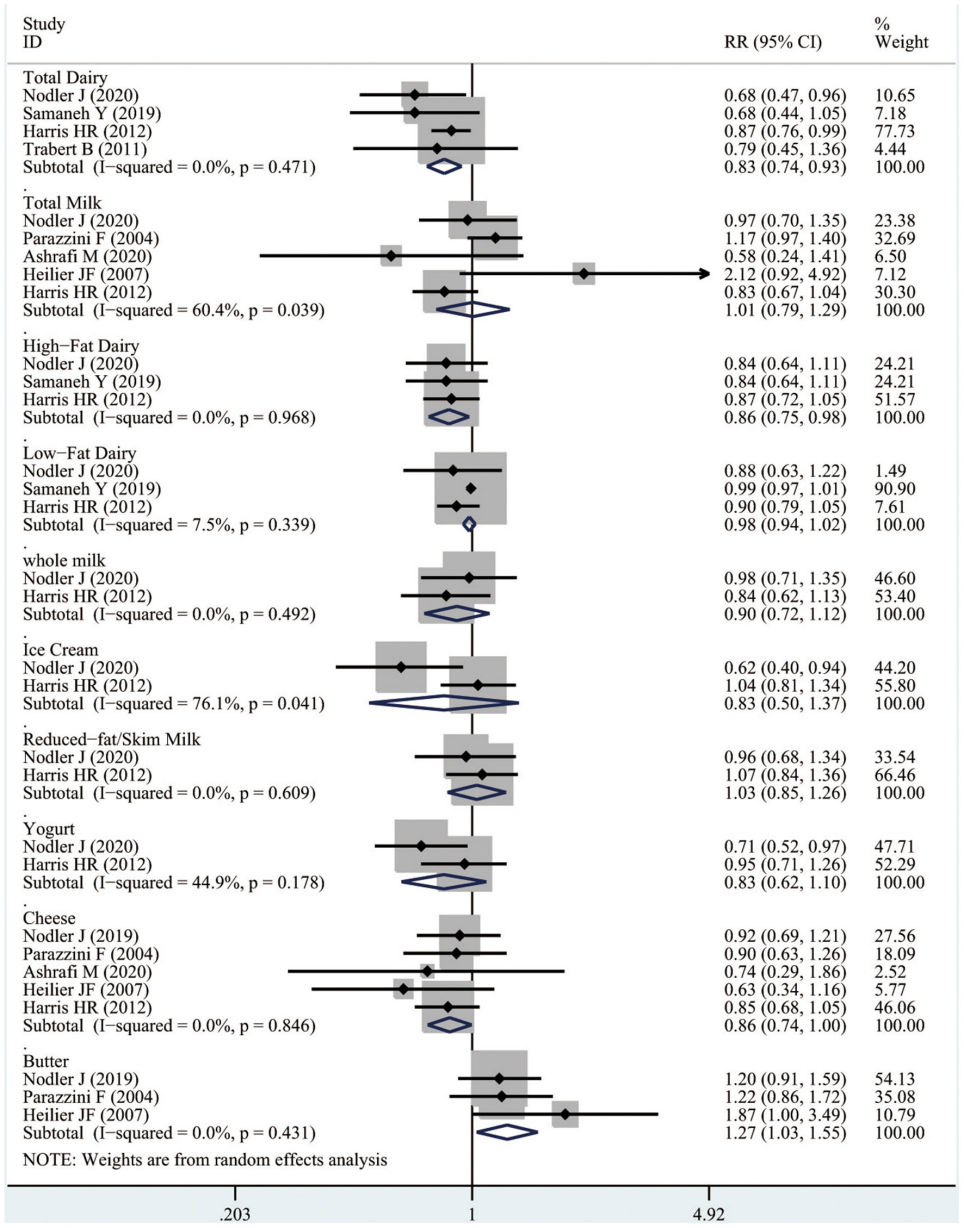
Forest plots of dairy products intake and the risk of endometriosis.

As shown in Figure 2, four studies involving 2,328 cases reported the association between total dairy products intake and the risk of endometriosis. Compared with the low dairy products intake, women with high dairy products would have a reduced risk of endometriosis (RR 0.83, 95% CI 0.74–0.93; *I*^2^ 0%).

**Figure 3:**
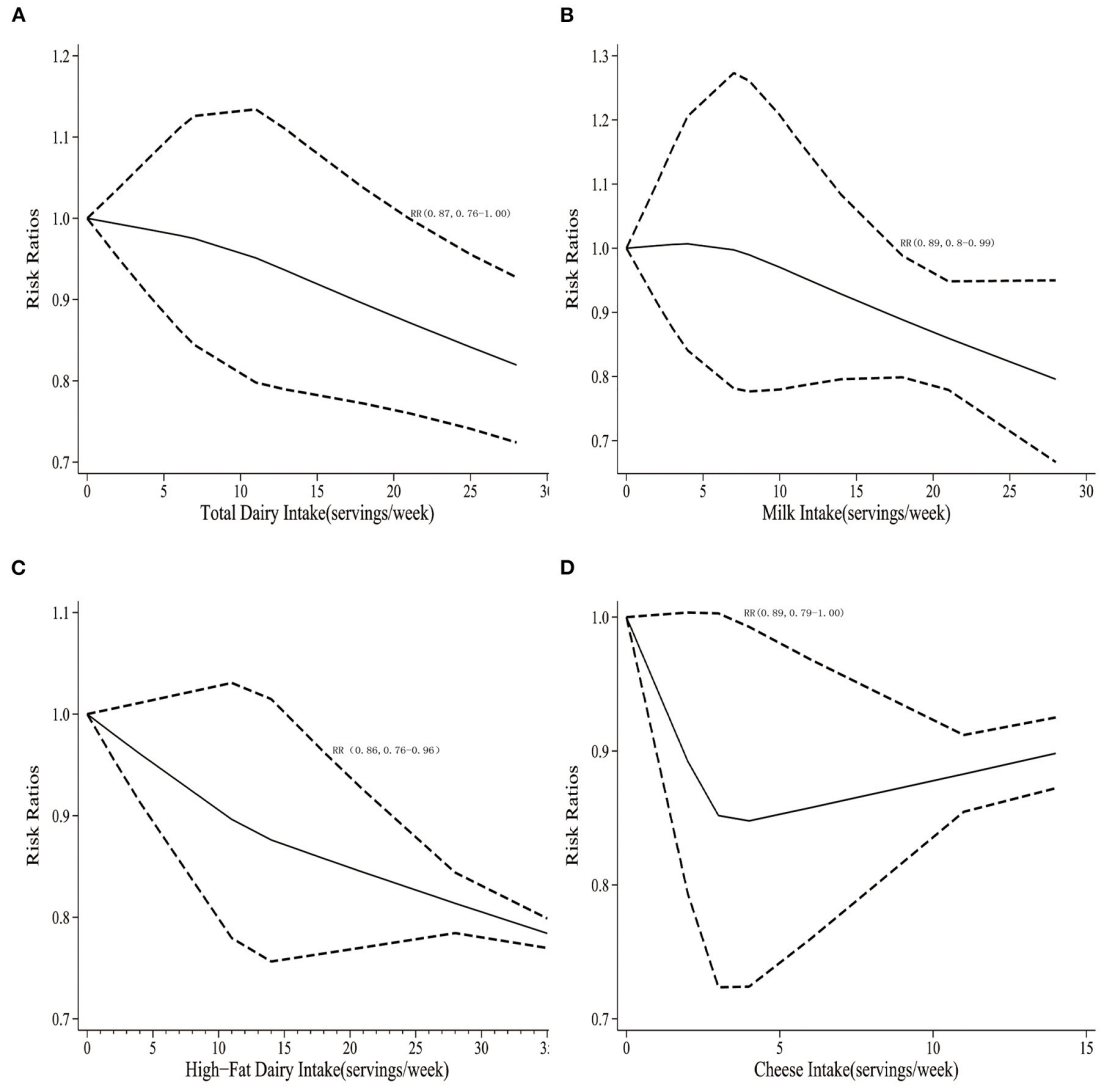
**(A)** Dose-response for total dairy products and the risk of endometriosis. **(B)** Dose-response for total milk products and the risk of endometriosis. **(C)** Dose-response for high-fat dairy products and the risk of endometriosis. **(D)** Dose-response for cheese and the risk of endometriosis.

Three studies containing a total of 2,250 cases were included in the dose-response meta-analysis. As illustrated in Figure 3A, total dairy intake was inversely associated with the risk of endometriosis. Specifically, as total dairy intake increased, the risk of endometriosis tended to decrease, with a significant decrease in the risk of endometriosis when dairy products intake was ≥21 servings/week (RR 0.87, 95% CI 0.76–1.00; *p*_non−linearity_ = 0.04).

#### Total Milk (High vs. Low)

Five studies involving 2,765 cases reported the association between milk product intake and the risk of endometriosis. No significant evidence suggested that high milk intake was associated with the risk of endometriosis comparing with the low milk intake (RR 1.01, 95% CI 0.79–1.29; *I*^2^ 0%).

Four studies containing a total of 2,677 cases were included in the dose-response meta-analysis. As shown in Figure 3B, milk intake tended to be inversely associated with the risk of endometriosis. Specifically, as milk intake increased, the risk of endometriosis would significantly decrease in the risk of endometriosis when milk consumption was ≥18 servings/week (RR 0.89, 95% CI 0.80–0.99; *p*_non−linearity_ = 0.022).

#### High-Fat Dairy (High vs. Low)

As illustrated in Figure 2, three studies including 2,044 cases suggested that females with high high-fat dairy intake might have a reduced risk of endometriosis compared to females with the low milk intake (RR 0.86, 95% CI 0.75–0.98; *I*^2^ 0%). Similarly, two studies containing a total of 1,966 cases were included in the dose-response meta-analysis, and the risk of endometriosis would significantly reduce when high-fat dairy consumption was ≥18 servings/week (RR 0.86, 95% CI 0.76–0.96; *p*_non−linearity_ = 0.012), as shown in Figure 3C.

#### Low-Fat Dairy (High vs. Low)

Three studies including 2,044 cases indicated that women with high low-fat dairy intake might not be associated with the reduce the risk of endometriosis comparing with the low-fat dairy intake (RR 0.98, 95% CI 0.94–1.02; *I*^2^ 0%), as illustrated in Figure 2. And yet due to the limited number of the current studies, the dose-response meta-analysis of low-fat dairy could not be conducted.

#### Whole Milk (High vs. Low)

Two studies including 1,966 cases indicated that no obvious positive evidence that high whole milk intake was associated with the risk of endometriosis comparing with the low whole milk intake (RR 0.90, 95% CI 0.72–1.12; *I*^2^ 0%), as illustrated in Figure 2. However, the dose-response meta-analysis for whole milk could not be conducted based on the limited current studies.

#### Ice Cream (High vs. Low)

Two studies including 1,966 cases revealed that no obvious association was found between the high ice cream intake and the risk of endometriosis comparing with the low ice cream intake (RR 0.83,95% CI 0.50–1.73; *I*^2^ 0%), as illustrated in Figure 2. However, the dose-response meta-analysis for whole milk could not be conducted based on the limited current studies.

#### Reduced-Fat/Skim Milk (High vs. Low)

Two studies including 1,966 cases indicated that there was no correlation was found between the high reduced fat/skim milk intake and the occurrence of endometriosis comparing with the low reduced fat/skim milk intake (RR 0.83, 95% CI 0.50–1.73; *I*^2^ 0%), as illustrated in Figure 2. However, the dose-response meta-analysis for reduced-fat/skim milk could not be conducted based on the limited current studies.

#### Yogurt (High vs. Low)

Two studies including 1,966 cases suggested that women with high yogurt intake might not have a reduced risk of endometriosis compared with women with low yogurt intake (RR 0.83, 95% CI 0.62–1.11; *I*^2^ 44.9%), as illustrated in Figure 2. However, the dose-response meta-analysis for yogurt could not be conducted based on the rare current studies.

#### Cheese (High vs. Low)

As shown in Figure 2, five studies involving 2,765 cases the association between cheese intake and the risk of endometriosis. Compared with the low cheese intake, females with high cheese intake might have a reduced risk of endometriosis (RR 0.86, 95% CI 0.74–1.00; *I*^2^ 0%).

Four studies containing a total of 2,677 cases were included in the dose-response meta-analysis. As illustrated in Figure 3D, cheese intake was inversely associated with the risk of endometriosis. Specifically, as cheese intake increased, the risk of endometriosis tended to have a significant decrease the risk of endometriosis when cheese intake was ≥2 servings/week (RR 0.89, 95% CI 0.79–1.00; *p*_non−linearity_ = 0.033).

#### Butter (High vs. Low)

Three studies including 1,173 cases suggested that females with high butter intake might have an increased risk of endometriosis comparing with the low butter intake (RR 1.27, 95% CI 1.03–1.55; *I*^2^ 0%), as illustrated in Figure 2. However, the dose-response meta-analysis for butter could not be performed due to the number of limited current studies.

The numbers of the studies that reported the association between specific dairy foods and the risk of endometriosis are so rare, therefore the subgroup analyses and the test and publication bias could not be conducted.

## Discussion

The present meta-analysis involving 120,706 participants showed that total dairy products intake would reduce the risk of endometriosis and there was a dose-dependent relationship, with a significant reduction in the risk of endometriosis when dairy intake was ≥21 servings/week. Similarly, the risk of endometriosis would significantly reduce when the intake of high-fat dairy products was ≥18 servings/week. However, in the qualitative analysis, this association was not found for low-fat dairy intake, and the dose-response could not be carried further due to the limited number of studies. Stratified analyses for specific dairy product categories indicated that high cheese intake might reduce the risk of endometriosis and there was a dose-dependent relationship. In the qualitative analysis, milk intake might not reduce the risk of endometriosis, but a dose-dependent relationship was found. Other specific dairy products such as whole milk, skim milk, ice cream, and yogurts have not shown significant evidence of an association with the development of endometriosis. Also, high butter intake would tend to increase the risk of endometriosis; no further dose-response could be performed to verify this result due to the limited number of studies.

The typical symptoms of endometriosis are pelvic pain, dysmenorrhea, dyspareunia, dysuria, malnutrition, and/or infertility (32). Women with endometriosis are often discovered because of dysmenorrhea, and the incidence of which is estimated to be between 45 and 90% in the developing countries (33, 34). Therefore, further improvement or treatment of the occurrence and progression of endometriosis remains an urgent challenge to be addressed. Current scientific evidence has pointed to the possibility that diet and lifestyle might affect inflammation, estrogen activity, the menstrual cycle, and prostaglandin metabolism in the body (35). As a result, diet and lifestyle can also affect the risk of endometriosis (36).

The pathogenesis of endometriosis remains inconclusive. Recently, a review by Rolla (37) systematically summarized the potential pathogenesis, diagnosis and treatment of endometriosis. Specifically, the current pathogenesis of endometriosis is mainly clustered on implantation theory, celomic theory, inflammatory disease, endometriomas, and hormonal receptors, with inflammatory receiving more attention from researchers. Scholl et al. (38) found increased expression of TNF-α in tissues from patients with endometriosis. Also, *in vitro*, TNF-α production by cultured endometrial cells were regulated by urocortin-2 and urocortin-3, neuropeptides expressed in human endometrium (39). Thus, TNF-α might be a key cytokine involved in the inflammatory aspects of endometriosis. In addition, IL-16, IL-8 in the peritoneal fluid may be involved in the pathogenesis of endometriosis by initiating or maintaining the inflammatory response in the peritoneal cavity (40). Cousins et al. suggested that human endometrium regenerates monthly in a cycle mediated by endometrial stem/progenitor cells such as CD140b^+^, CD146^+^, or SUSD2^+^ endometrial mesenchymal stem cells (eMSCs). N-cadherin^+^ endometrial epithelial progenitor cells and borderline population cells might be involved in the progression of the disease (41). Besides, it has been shown that Vitamin D receptor (VDR) and 1α-hydroxylase were overexpressed in the endometrium of patients with endometriosis, and thus Vitamin D might mediate the immune mechanism of endometriosis pathogenesis; however, this findings lacked evidence to support the relationship between VDR gene polymorphisms and endometriosis (42, 43). In a study by Zemel et al. (9) it was shown that in a mouse model, dairy diet reduced markers of oxidative and inflammatory stress, including TNF-αinhibitor and IL-6, which might laterally explain the possibility of dairy products reducing the risk of endometriosis (44). Besides, dairy products are high in magnesium, which not only reduces levels of inflammatory markers such as IL-6 and TNFα-R2 but also relaxes smooth muscle and might reduce retrograde menstruation (9). Our study showed that a total dairy products intake of more than 21 servings/week, or an average of >3 servings/day, significantly reduced the risk of endometriosis by 13% compared to women with no or low dairy intake, which is similar to the findings of Harris et al. (29). Simultaneously, the study by Harris et al. showed that milk intake reduced the risk of endometriosis, but only in the group with more than 2 servings/day. Likewise, our meta-analysis was conducted through High vs. Low showing that no support for the conclusion that milk reduces endometriosis, as this generalized between-group comparison could potentially attenuate the benefit of milk, whereas the dose-response analyses showed a reduced risk of endometriosis when milk intake was more than 18 servings/week, which is similar to the findings of Harris et al. (29). High-fat dairy products rich in calcium and vitamin D reduces oxidative stress and inflammatory stress, thereby reducing the risk of endometriosis (45). Dietary structure is a very complex lifestyle, and the underlying mechanisms of diet and the risk of endometriosis still need further research. Importantly, current guidelines for the diagnosis and management of endometriosis vary greatly in terms of recommendations and methodological quality from different countries to institutions, and more high-quality studies are needed to further refine them in the future (46).

Notably, our meta-analysis has the following advantages. First, to the best of our knowledge, this is the first study to systematically perform a qualitative meta-analysis of the relationship between dairy product intake and endometriosis. Furthermore, the qualitative results of the meta-analysis are further validated by the presentation of a dose-response meta-analysis. Second, some of the results of this study are contradictory to what is currently known, which will provide direction for future studies. Third, inter-study heterogeneity was low to moderate, and the quality of the included studies is high and the results are reliable.

Inevitably, this study also has some limitations. Firstly, due to the limited number of existing studies, subgroup analysis, and publication bias cannot be carried out. Second, although the results of most studies were adjusted for maximum covariates, the effects of residual confounding variables were not excluded. Third, due to the limited number of studies available, some specific types of dairy products, such as low-fat dairy, ice cream, and yogurt were not available for dose-response analyses.

## Conclusion

Overall, dairy products intake is associated with a reduction in endometriosis, with significant effects when the average daily intake ≥3 servings. When analyzed according to the specific type of dairy product, it was shown that females with higher high-fat dairy and cheese intake might have a reduced risk of endometriosis. However, high butter intake might be associated to the increased risk of endometriosis. More future studies are needed to validate and add to this finding.

## Data Availability Statement

The original contributions presented in the study are included in the article/Supplementary Material, further inquiries can be directed to the corresponding author.

## Author Contributions

XQ, MG, and QS designed the study. XL, LP, and WC did the literature searches and designed the data extraction form. XL, XQ, WC, MG, and QS collected the data. LP, WC, and WZ did the statistical analyses. XL, LP, WC, and WZ supervised the entire project. All authors critically revised subsequent drafts, read, and approved the submitted manuscript.

## Conflict of Interest

The authors declare that the research was conducted in the absence of any commercial or financial relationships that could be construed as a potential conflict of interest.
