# Supplementary material for: Relationship Between Dairy Products Intake and Risk of Endometriosis: A Systematic Review and Dose-Response Meta-Analysis
Source: Front Nutr. 2021 Jul 22;8:701860. doi: 10.3389/fnut.2021.701860 (PMC8339299; doi:10.3389/fnut.2021.701860)
Supplement: Supplementary file 1 [file Data_Sheet_1.docx]

***Appendix 1***

**Pubmed**

1. "dairy products"[MeSH Terms] OR ("dairy"[All Fields] AND "products"[All Fields]) OR "dairy products"[All Fields] OR ("dairy"[All Fields] AND "product"[All Fields]) OR "dairy product"[All Fields] OR ("dairy products"[MeSH Terms] OR ("dairy"[All Fields] AND "products"[All Fields]) OR "dairy products"[All Fields] OR ("product"[All Fields] AND "dairy"[All Fields]) OR "product dairy"[All Fields]) OR ("dairy products"[MeSH Terms] OR ("dairy"[All Fields] AND "products"[All Fields]) OR "dairy products"[All Fields] OR ("products"[All Fields] AND "dairy"[All Fields]) OR "products dairy"[All Fields]) OR ("milk, human"[MeSH Terms] OR ("milk"[All Fields] AND "human"[All Fields]) OR "human milk"[All Fields] OR "milk"[All Fields] OR "milk"[MeSH Terms])

2. "endometriosis"[MeSH Terms] OR "endometriosis"[All Fields] OR "endometrioses"[All Fields] OR "endometriosis"[MeSH Terms] OR "endometriosis"[All Fields] OR "endometrioma"[All Fields] OR "endometriomas"[All Fields] OR "endometriosis"[MeSH Terms] OR "endometriosis"[All Fields] OR "endometrioma"[All Fields] OR "endometriomas"[All Fields]

3.1 and 2 37

**Embase**

1. 'dairy product'/exp
2. dairy AND products OR (milk AND product) OR ('milk derived' AND product) OR ('milk derived' AND products) OR milk
3. 1 or 3
4. 'endometriosis'/exp
5. appendix AND endometriosis OR (endometriosis AND externa)
6. #5 OR #6
7. 3 and 6 61

**Cochrane**

1. MeSH descriptor: [Endometriosis] explode all trees
2. (Endometrioses) OR (Endometriomas) OR (Endometrioma)
3. #1 or #2
4. MeSH descriptor: [Dairying] explode all trees
5. (Product, Dairy) OR (Dairy Product) OR (Products, Dairy)
6. #4 or #5
7. 3 and 6 0

**Web of Science**

1. TOPIC: (Endometriosis) OR TOPIC: (Endometrioses) OR TOPIC: (Endometrioma) OR TOPIC: (Endometriomas)
2. TOPIC: (Dairy Products) OR TOPIC: (Dairy Product) OR TOPIC: (Product, Dairy) OR TOPIC: (Products, Dairy)
3. 1 and 2 344
