# Supplementary material for: Relationship Between Dairy Products Intake and Risk of Endometriosis: A Systematic Review and Dose-Response Meta-Analysis
Source: Front Nutr. 2021 Jul 22;8:701860. doi: 10.3389/fnut.2021.701860 (PMC8339299; doi:10.3389/fnut.2021.701860)
Supplement: Supplementary file 2 [file Table_1.docx]

| ***Supplementary Table 1. Quality Assessment of the 7 observational Studies*** | | | | |
| --- | --- | --- | --- | --- |
| **Study**  **(First Author, Year)** | **Selection** | **Comparability** | **Outcome/ exposure** | **Total** |
| Nodler J,2020 | **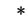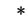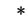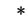** | **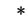** | **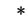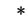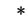** | **8** |
| Parazzini F,2004 | **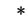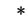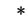** | **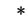** | **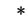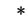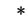** | **7** |
| Samaneh Y ,2019 | **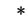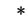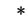** | **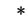** | **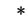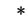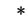** | **7** |
| Ashrafi M,2020 | **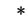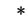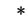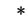** | **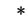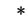** | **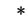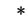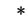** | **8** |
| Harris HR,2012 | **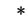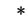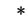** | **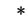** | **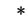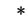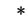** | **7** |
| Trabert B,2011 | **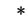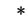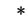** | **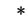** | **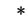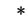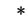** | **7** |
| Heilier JF,2007 | **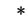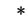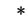** | **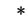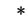** | **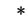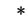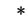** | **8** |
